# Chromatin-associated cullin-RING E3 ubiquitin ligases: keeping transcriptionally active NF-κB in check

**DOI:** 10.3389/fimmu.2025.1584999

**Published:** 2025-04-16

**Authors:** Mengyao Gong, Junqi Luo, Qiankun Liang, Yi Liu, Yuejuan Zheng, Xiao-Dong Yang

**Affiliations:** ^1^ The Research Center for Traditional Chinese Medicine, Shanghai Institute of Infectious Diseases and Biosecurity, Shanghai University of Traditional Chinese Medicine, Shanghai, China; ^2^ Center for Traditional Chinese Medicine and Immunology Research, School of Integrative Medicine, Shanghai University of Traditional Chinese Medicine, Shanghai, China

**Keywords:** E3 ligase, ubiquitination, NF-κB, RelA, chromatin, transcription, inflammation

## Abstract

Nuclear factor-κB (NF-κB) constitutes a family of transcription factors that serve as a critical regulatory hub, dynamically orchestrating inflammatory and immune responses to maintain homeostasis and protect against pathogenic threats. Persistent activation of NF-κB has been implicated in the pathogenesis of various inflammatory diseases and cancer. A critical mechanism to prevent excessive inflammation and its harmful effects is the timely termination of NF-κB’s transcriptional activity on target genes. This termination can be facilitated through the ubiquitination and subsequent proteasomal degradation of chromatin-bound RelA, the most active subunit of NF-κB. Several multi-subunit cullin-RING E3 ubiquitin ligases, composed of elongin B/C, cullin2/5, and SOCS-box proteins, have been identified to target RelA for degradation. These E3s, known as ECS complexes, use SOCS-box proteins as substrate-recognizing subunits to engage RelA. SOCS1 is the first identified SOCS-box member that functions in ECS^SOCS1^ to target chromatin-bound RelA for ubiquitination. Specifically, SOCS1 collaborates with accessory proteins COMMD1 and GCN5 to preferentially recognize Ser468-phosphorylated RelA. Our recent work demonstrates that WSB1 and WSB2 (WSB1/2), two additional SOCS-box proteins with structurally similar WD40 repeat domains, function as substrate-recognizing subunits of ECS^WSB1/2^ to specifically mediate the ubiquitination and degradation of chromatin-associated RelA methylated at Lys314/315. In this review, we summarize the discovery and functional importance of ECS^SOCS1^ and ECS^WSB1/2^ in terminating NF-κB activity, highlight the distinct molecular mechanisms by which they ubiquitinate chromatin-associated RelA in a modification- and gene-specific manner, and discuss their potential as therapeutic targets for inflammatory diseases and cancer.

## 1 Introduction

The nuclear factor-κB (NF-κB) family of transcription factors is a master regulator controlling the expression of a variety of genes involved in inflammatory and immune responses for defense against pathogens and injuries. It is of paramount importance that NF-κB action is eventually terminated to avert persistent inflammation, a condition that has been associated with the development and progression of a myriad of diseases, such as arthritis, septic shock, and cancer (1, 2).

Five NF-κB subunits, p50, p52, RelA (p65), RelB and c-Rel assemble into hetero- or homo-dimers in a context-dependent manner with the RelA/p50 heterodimer being the most common form functioning in the classical NF-κB pathway (3, 4). The activation of NF-κB is governed by a series of sophisticated spatiotemporal mechanisms. In unstimulated cells, NF-κB is associated with the inhibitor of κB (IκB) proteins in the cytosol and remains inactive. Upon stimulation by agents such as TNFα or lipopolysaccharide (LPS), signals are transduced through cognate receptors on the cell surface to activate the IκB kinase (IKK) complex which in turn phosphorylates IκBs, triggering their proteasomal degradation. Thus, the freed NF-κB can translocate into the nucleus, where it binds κB site-containing regions of chromatin to initiate gene transcription (3, 4).

NF-κB-mediated transcription is a self-limiting process, and several sophisticated mechanisms have been discovered to ensure proper termination of transcriptionally active NF-κB (1, 5). First, IκBs are target genes of NF-κB. The resynthesized IκBs can disassociate NF-κB from chromatin and relocate it back to the cytosol (3, 4). In addition to IκBs, other negative feedback regulators, such as A20 and CYLD, are also induced by active NF-κB to suppress upstream signaling events that contribute to sustained activation of NF-κB (6–8). Alternatively, it has been demonstrated that following NF-κB activation, ubiquitination and proteasomal degradation of chromatin-bound RelA is a crucial complementary mechanism for the timely termination of NF-κB-dependent transcription of a variety of genes (9–14).This termination mechanism is further supported by the findings that deubiquitination of chromatin-bound RelA by USP7 stabilizes RelA and promotes NF-κB-dependent transcription (15, 16). To date, two types of E3 ubiquitin ligases targeting chromatin-bound RelA have been identified, both of which are multi-subunit cullin-RING ubiquitin ligases (CRLs).

## 2 CRL E3s targeting chromatin-bound RelA

CRLs constitute the largest subfamily of RING-type E3 ubiquitin ligases, with over 200 members that are estimated to be responsible for approximately 20% of all ubiquitination in cells (17). In canonical CRLs, cullins serve as central scaffolding proteins with their C-terminal domains engaging a specific RBX protein, RBX1 (associated with Cul1–4) or RBX2 (associated with Cul5) and their N-terminal domain binding a cullin-specific substrate receptor via one or more adaptor proteins. Notably, Cul2 and Cul5 are distinct among cullins because they complex with elongin B/C as adaptor proteins and a SOCS box protein as a substrate receptor (18, 19). In this scenario, it is the SOCS box protein that brings a substrate to the E3 complex, thereby determining the substrate specificity.

**Figure 1:**
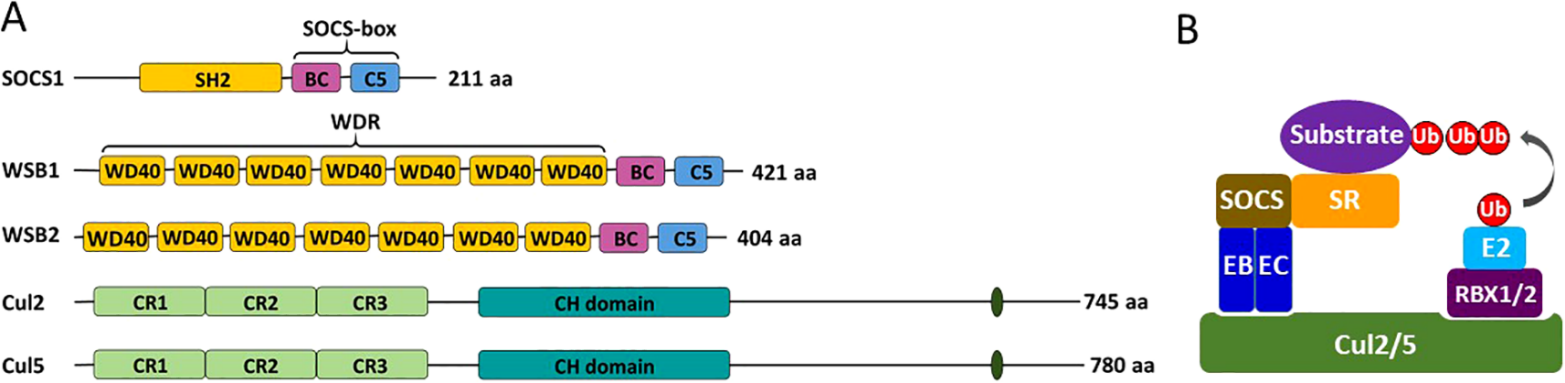
Domain structure of key components and assembly of the ECS^SOCS1^ and ECS^WSB1/2^. **(A)** Domain structure of SOCS1, WSB1, WSB2, Cul2, and Cul5 are drew roughly to scale. **(B)** Cul2/5 each serves as a core scaffold that assembles with the catalytic component RBX1/2 and the adapter elongin B/C, which recruit an E2 enzyme and a SOCS-box protein via binding the SOCS-box domain, respectively. Eventually the SOCS-box protein recruits a substrate protein via the substrate-recognizing (SR) domain for the transfer of ubiquitin (Ub) to the substrate from the E2. SH2, SRC homology 2 domain; BC, BC box; C5, Cul5 box; WD40, WD40 repeat; CR, cullin repeat; CH, cullin homology domain; EB, Elongin B; EC, Elongin C; E2, ubiquitin-conjugating enzyme.

Typical SOCS-box proteins feature diverse N-terminal domains and a highly conserved C-terminal SOCS box, which consists of a BC box followed by a Cul5 box that mediate the interactions with elongin C and Cul5, respectively (20) (**Figure 1**). Interestingly, SOCS1 represents an exception within this paradigm. A key residue in the Cul5 box consensus sequence (LPϕP), Pro184, is replaced by an asparagine residue in SOCS1 (21), enabling SOCS1 interact with Cul2 rather than Cul5 (10). Based on their N-terminal domains, which mediate substrate binding, typical SOCS-box proteins can be categorized into four groups: 1) SH2 domain-containing members (SOCS1-7 and CIS), 2) ankyrin domain-containing members (ASB1-18), 3) SPRY domain-containing members (SPSB1-4), and 4) WD40 domain-containing members (WSB1-2) (22). For majority of these SOCS-box proteins, the specific substrates they target for ECS-mediated ubiquitination remain poorly characterized.

### 2.1 ECS^SOCS1^ targets phosphorylated RelA

The identification of ECS^SOCS1^ as the first CRL E3 targeting chromatin-associated RelA for ubiquitination and degradation was initiated by the studies of COMMD1, the prototype of the copper metabolism gene MURR1 domain (COMMD)-containing family of proteins involved in suppressing NF-κB function via different means (23). In contrast to other members of this family, COMMD1 neither regulates TNFα-induced nuclear translocation of RelA from the cytosol nor modulates NF-κB’s ability to bind DNA, while it strongly interacts with RelA’s Rel homology domain which is responsible for binding of DNA and IκBs. Interestingly, upon TNFα stimulation, COMMD1 is recruited to the promoter of an NF-κB target gene, where it down-regulates the level of DNA-bound RelA through an unknown mechanism (23). Subsequent studies demonstrated that COMMD1 destabilizes RelA by promoting TNFα-stimulated ubiquitination and degradation of RelA. Moreover, COMMD1 was shown to physically interact with components of the E3 complex ECS^SOCS1^, elongins B and C, Cul2 and SOCS1. Further research revealed that COMMD1 enhances the interaction between SOCS1 and RelA, thereby promoting ECS^SOCS1^-dependent ubiquitination of RelA (10).

In general, CRL-mediated ubiquitination of a substrate requires a prior modification, typically phosphorylation, to enable recognition by the substrate receptor. Although SOCS1 has an SH2 domain, the most prevalent protein-binding module recognizing a phosphotyrosine residue (22, 24), there is no evidence that tyrosine phosphorylation of RelA is involved in the SOCS1-dependent ubiquitination. Intriguingly, IKK-dependent phosphorylation of RelA at serine (S) 468 is a prerequisite for binding to ECS^SOCS1^, as evidenced by the TNFα-induced interactions of RelA with COMMD1 and Cul2. Consistent with this, mutation of S468 to alanine dramatically reduced the binding of COMMD1 and Cul2 to RelA and correspondingly diminished the ubiquitination and degradation of RelA (9). Supporting this finding, an independent study also highlighted the importance of S468 phosphorylation of RelA for ECS^SOCS1^-mediated ubiquitination (11). This study revealed that in this process another accessory protein GCN5, a histone acetyltransferase, associated with COMMD1 and RelA and promoted ubiquitination and degradation of RelA in S468 phosphorylation-dependent manner. However, the acetyltransferase activity of GCN5 was dispensable for this function (11), suggesting that GCN5 serves as a scaffold in this E3 complex.

The role of ECS^SOCS1^ in the pathogenesis of inflammatory diseases has been partially confirmed in COMMD1 knockout mouse models. In studies examining the role of COMMD1 in LPS- or cecal ligation and puncture (CLP)-induced sepsis and DSS-induced colitis models, mice with a specific disruption of *Commd1* in myeloid cells exhibited much stronger inflammatory responses, characterized by overproduction of proinflammatory cytokines and higher disease severity, compared to their wild-type littermates (25). In line with these observations, deficiency of myeloid *Commd1* exacerbated HFC diet-induced liver inflammation that is largely dependent on NF-κB action (26).

Despite the importance of ECS^SOCS1^-dependent degradation of S468 phosphorylated RelA, replacement of S468 with a non-phosphorylatable Alanine residue only partially rescued but did not fully prevent the degradation of RelA (11). This finding implies the existence of ECS^SOCS1^-independent mechanism(s) for the degradation of chromatin-bound RelA.

### 2.2 ECS^WSB1/2^ targets methylated RelA

Around the time when S468 phosphorylation-dependent RelA ubiquitination by ECS^SOCS1^ was discovered, we found that TNFα or LPS stimulation induced mono-methylation (designated as methylation for simplicity thereafter) of RelA at lysines 314 and 315 (K314/315) by the histone lysine methyltransferase Son promoters of some NF-κB target genes, such as *IL-6* and *IL-8* (13, 27). We further demonstrated that, like S468 phosphorylation, K314/315 methylation is also involved in ubiquitination and degradation of RelA (13, 27). These findings provide early evidence that methylation can prime a protein for ubiquitination and degradation, a previously uncharacterized regulatory mechanism (28). Mutation of K314/315 to Arginine residues or depletion of Set9 prolonged the stay of RelA on chromatin and enhanced the expression of NF-κB target genes (13). These observations led us to hypothesize that there may exist E3 ubiquitin ligase(s) capable of recognizing K314/315-methylated RelA to induce degradation through ubiquitination.

To identify such an E3 ligase or a substrate receptor of a multi-subunit E3 complex, we designed a cell-based assay to screen among SOCS-box proteins (14). This approach was motivated by two key considerations: First, as implied by their name, SOCS-box proteins are typically involved in negatively regulating cytokine-induced signaling pathways, in which NF-κB is frequently activated. Second, several SOCS-box proteins have been shown to induce RelA degradation, including SOCS1, which has been identified as a substrate receptor of ECS^SOCS1^ targeting chromatin-associated RelA (10). In this assay, each of SOCS-box proteins was co-expressed with Set9 and either wild-type (WT) RelA or a methylation-deficient RelA mutant (K314315R), and the levels of RelA were assessed by immunoblotting. Among the SOCS-box proteins tested, only WSB1 and WSB2 markedly reduce the level WT RelA but not the methylation-deficient mutant, suggesting that WSB1 and WSB2 promote RelA degradation in a manner dependent on K314/315 methylation (14).

Notably, WSB1 and WSB2 share a similar bipartite domain structure, featuring a WD40 repeat (WDR) domain at the N-terminus and a SOCS box domain at the C-terminus (**Figure 1A**). Alignment analysis of the AlphaFold2-predicted 3D structures of WSB1 and WSB2 confirmed that their overall structures are highly similar, with their WDRs characterized by a seven-bladed β-propeller structure (14). This structural feature is reminiscent of the classical WDR domains in other proteins, such as WDR5, whose WDR domain has been shown to specifically recognize lysine-methylated histone H3 (29). The shared structural feature between WSB1/2 and WDR5 further supports the hypothesis that WSB1 and WSB2 can target K314/315-methylated RelA.

To test this hypothesis, a series of functional experiments were conducted. Denaturing ubiquitination assays revealed that upon TNFα stimulation, both WSB1 and WSB2 could induce ubiquitination of chromatin-bound RelA, with WSB2 appearing to play a more dominant role than WSB1 in the cell types tested. Importantly, mutation of K314/315 to arginine residues significantly reduced WSB1- and WSB2-dependent RelA ubiquitination (14). RNA-sequencing analysis of control and WSB2 knockdown cells demonstrated that depletion of WSB2 enhanced the expression of dozens of TNFα-induced genes, most of which were NF-κB target genes, including those encoding pro-inflammatory cytokines and chemokines (14). Furthermore, chromatin immunoprecipitation (ChIP) assays showed that TNFα stimulation increased the recruitment of WSB2 to the promoters of WSB2-regulated NF-κB target genes, where the occupancy of both total and K314/315-methylated RelA was elevated upon WSB2 depletion (14). Collectively, these findings reveal that during TNFα-induced NF-κB activation, WSB1/2 are crucial for degrading transcriptionally active RelA that is marked by prior methylation of K314/315. Given that WSB1/2 had been shown to form CRL E3 complexes with Cul5 and elongin B/C (30), we designated these E3 complexes as ECS^WSB1/2^.

In efforts to define the molecular mechanism by which WSB1/2 recognize methylated RelA, GST pulldown assays were initially performed to examine the binding of GST-fused WSB1/2 to RelA in lysates from transfected cells, as well as the effect of K314/315 methylation. It turned out that both WSB1 and WSB2 could bind to RelA in a manner that was largely dependent on K314/315 methylation. This binding capacity relied on the WDR domain rather than the SOCS box domain of WSB1/2 (14). More importantly, a subsequent GST pulldown experiment using GST-fused WDR domain of WSB2 and short RelA peptides containing K314/315 demonstrated that the WSB2 WDR domain could directly bind to the unmodified peptide, albeit weakly. However, methylation of K314/315 significantly enhanced this binding (14). This finding provides compelling evidence that WSB2 preferentially binds to RelA when K314/315 are methylated.

To elucidate how K314/315 methylation enables the robust interaction between WSB1/2 and RelA, we employed the latest computational modeling techniques in conjunction with the reported crystal structure of the WDR5 WDR domain complexed with a lysine-methylated histone H3 peptide (H3K4me2). Modeling with RelA peptides and the WSB2 WDR domain implied that the aspartic acid (D) at position 158 in this WDR domain, which is conserved in WSB1 (D175) and WDR5 (D92), coordinates K314 or K315 of RelA. Methylation of either lysine residue creates a higher binding affinity for WSB2 WDR domain (14). Additionally, we identified an interaction between the side chain of K314 and glutamate (E) 28 in the WSB2 WDR domain, mediated by a bridging water molecule in the methylated context. This interaction is analogous to the water-mediated interaction between H3K4me2 and E322 in WDR5 (28). Experiments with WSB2 variants harboring point mutations confirmed that, compared to WT WSB2, substitution of D158 or E28 with an alanine residue diminished the ability of WSB2 to bind and ubiquitinate methylated RelA (14). Collectively, these findings delineate the detailed molecular mechanism by which WSB1/2 preferentially target methylated RelA for ubiquitination and degradation.

## 3 The differences between ECS^SOCS1^ and ECS^WSB1/2^

**Figure 2:**
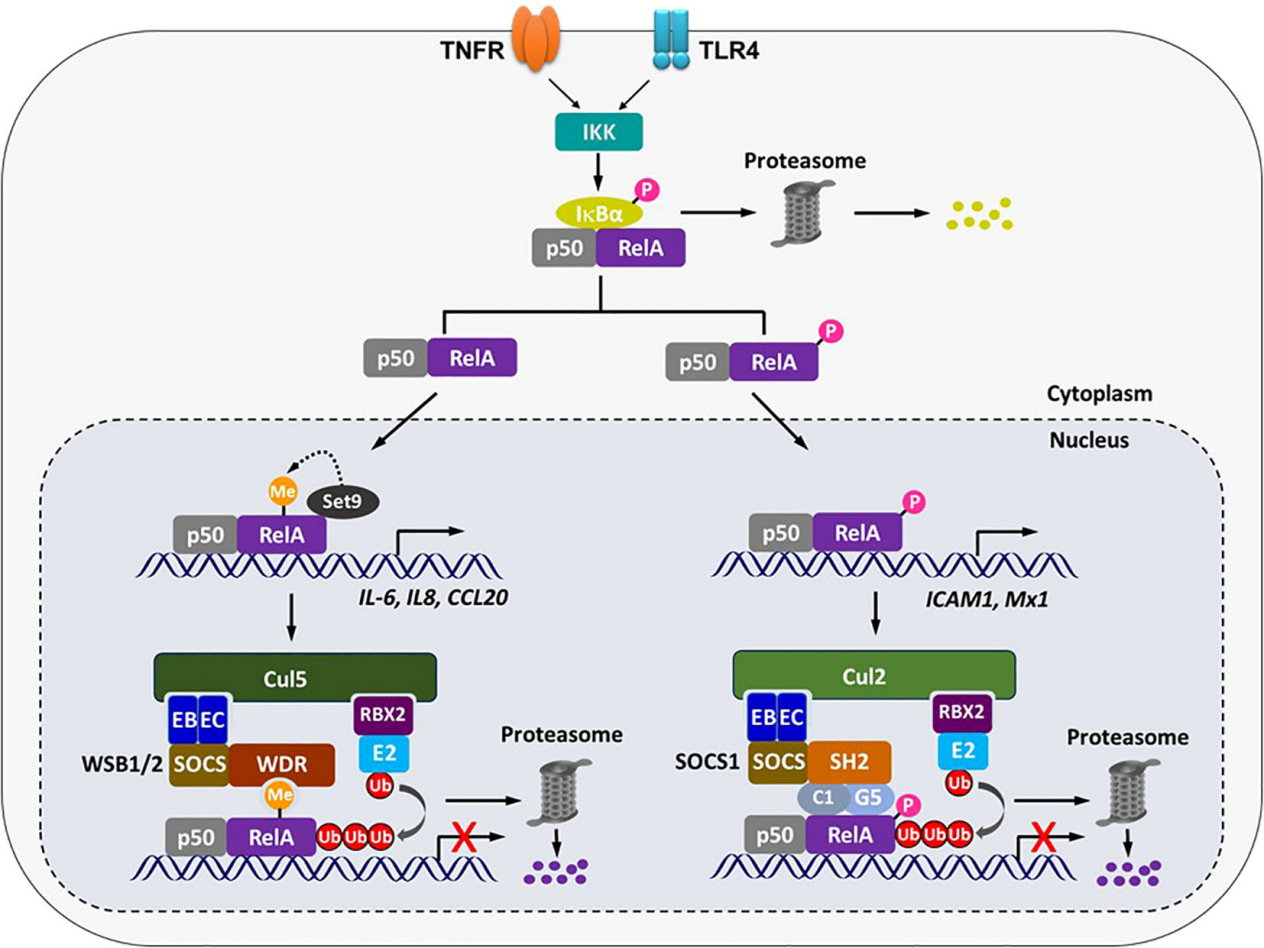
ECS^SOCS1^ and ECS^WSB1/2^ contribute to the degradation of chromatin-associated NF-κB subunit RelA in modification- and gene-specific manner. In response to signals activating TNFR and TLR4, IKK is activated and it then phosphorylates IκBα for degradation. Some portion of RelA is also subject to phosphorylation (P) by IKK before NF-κB’s translocation into the nucleus. In the nucleus, phosphorylated form of RelA binds to promoters of a subset of NF-κB-dependent genes where it can be targeted by SOCS1-COMMD1 (C1)-GCN5 (G5) -containing ECS^SOCS1^ for ubiquitination (Ub) and proteasomal degradation. Other form, likely unphosphorylated, of RelA can be lysine-methylated by Set9 on another subset of NF-κB-dependent genes, and this methylation (Me) is recognized by the WDR domain of WSB1 or WSB2 which functions as a substrate receptor of ECS^WSB1/2^ for ubiquitination and proteasomal degradation of RelA. EB, Elongin B; EC, Elongin C; E2, ubiquitin-conjugating enzyme.

To date, ECS^SOCS1^ and ECS^WSB1/2^ are the best-characterized CRL E3 ligases that target chromatin-bound RelA for termination of transitionally active NF-κB. Although both E3 complexes are composed of Cullin/RBX as the core scaffold, elongin B/C as adaptors, and a SOCS-box protein as the substrate receptor, and both function as negative regulatory loops to limit NF-κB activity on chromatin, they differ in at least the following four aspects (**Figure 2**). 1) WSB1 or WSB2 alone appears to be sufficient for binding to RelA (14), with no evidence suggesting the requirement for additional proteins. In contrast, SOCS1 requires accessory proteins COMMD1 and GCN5 to facilitate the association of ECSSOCS1 with RelA; depletion of either COMMD1 or GCN5 dramatically reduces the ability of ECS^SOCS1^ to ubiquitinate RelA (9–11). 2) ECS^WSB1/2^ preferentially bind to K314/315-methylated RelA via their WDR domains through a conserved mechanism (14). However, ECSSOCS1 binds more effectively to S468-phosphorylated RelA. The precise mechanism by which SOCS1, COMMD1, and GCN5 sense this phosphorylation, either individually or in combination, remains to be elucidated (9, 11). 3) ECS^WSB1/2^ mainly targets chromatin-bound RelA since solely this form of RelA can be K314/315-methylated (13, 14, 27). In addition to chromatin-bound RelA, ECS^SOCS1^ was also shown to target nuclear chromatin-unbound RelA (11). This is plausible because S468-phosphorylation of RelA is mediated by cytoplasmic kinases, IKKα/β/ϵ (9, 11), and supposedly occurs before the nuclear translocation. Therefore, once in the nucleus, S468-phosphorylated RelA could either associate with chromatin or remain unbound. 4) These two E3 complexes regulate distinct yet overlapping sets of NF-κB target genes. RNA-seq and RT-PCR analyses indicate that WSB1/2 suppress cytokine and chemokine genes, such as *IL1B*, *IL6*, *IL8*, *CCL20* and *CXCL3.* Notably, at least two of them, *IL6* and *IL8*, were previously shown in our studies to be repressed by Set9-mediated RelA methylation (13, 14, 27). As for ECS^SOCS1^, the regulation of gene expression by different components seem to vary. *ICAM1* is a representative gene that can be inhibited by COMMD1, GCN5 or RelA S468 phosphorylation (9–11). However, it has been shown that COMMD1 and GCN5 can differently down-regulate other NF-κB target genes, including *CXCL1, CCL2* and *TNFα* (10, 11). Thus, these two types of E3 complexes may play non-redundant roles in terminating NF-κB-dependent transcription by targeting chromatin-bound RelA in a gene- and/or RelA modification-specific manner.

## 4 Conclusion and future directions

Accumulating evidence has demonstrated that the two CRL E3 complexes, ECS^SOCS1^ and ECS^WSB1/2^, play critical and non-redundant roles in terminating NF-κB transcriptional activity by targeting chromatin-bound RelA for ubiquitination and proteasomal degradation. These findings underscore the potential of ECS^SOCS1^ and ECS^WSB1/2^ as therapeutic targets for the treatment of inflammatory disorders and cancer. Specifically, upregulating the components or enhancing the assembly of these E3 complexes holds significant promise, which has been exemplified by the recent discovery of an antitumor peptide CIGB-552 (31, 32). This peptide exerts antiproliferative and cytotoxic effects on cancer cells by interacting with and stabilizing COMMD1, thereby inhibiting the expression of NF-κB-regulated antiapoptotic genes (31, 32).

Despite substantial progress in this field, several critical questions remain to be addressed. For example, the mechanisms by which SOCS1, COMMD1, and GCN5 assemble to interact more effectively with RelA, and how S468 phosphorylation facilitates RelA’s association with the SOCS1-COMMD1-GCN5 complex, are still poorly understood. Additionally, it remains unclear whether WSB1/2 function similarly *in vivo* within animal models of inflammatory diseases as they do in cellular contexts as substrate receptors of ECS^WSB1/2^. Finally, during NF-κB activation, the factors that determine whether RelA undergoes IKK-mediated S468 phosphorylation or Set9-dependent K314/315 methylation, and whether there is any crosstalk between these two modifications and the corresponding CRL E3 ligases, await further investigation.
